# Effect of fermentation time on the content of bioactive compounds with cosmetic and dermatological properties in Kombucha Yerba Mate extracts

**DOI:** 10.1038/s41598-021-98191-6

**Published:** 2021-09-22

**Authors:** Aleksandra Ziemlewska, Zofia Nizioł-Łukaszewska, Tomasz Bujak, Martyna Zagórska-Dziok, Magdalena Wójciak, Ireneusz Sowa

**Affiliations:** 1 Department of Technology of Cosmetic and Pharmaceutical Products, University of Information Technology and Management in Rzeszow, Rzeszow, Poland; 2 Department of Analytical Chemistry, Medical University of Lublin, Lublin, Poland

**Keywords:** Cell biology, Plant sciences

## Abstract

Kombucha is a beverage made by fermenting sugared tea using a symbiotic culture of bacteria belonging to the genus *Acetobacter, Gluconobacter*, and the yeasts of the genus *Saccharomyces* along with glucuronic acid, which has health-promoting properties. The paper presents the evaluation of ferments as a potential cosmetic raw material obtained from Yerba Mate after different fermentation times with the addition of Kombucha. Fermented and unfermented extracts were compared in terms of chemical composition and biological activity. The antioxidant potential of obtained ferments was analyzed by evaluating the scavenging of external and intracellular free radicals. Cytotoxicity was determined on keratinocyte and fibroblast cell lines, resulting in significant increase in cell viability for the ferments. The ferments, especially after 14 and 21 days of fermentation showed strong ability to inhibit (about 40% for F21) the activity of lipoxygenase, collagenase and elastase enzymes and long‐lasting hydration after their application on the skin. Moreover, active chemical compounds, including phenolic acids, xanthines and flavonoids were identified by HPLC/ESI–MS. The results showed that both the analyzed Yerba Mate extract and the ferments obtained with Kombucha may be valuable ingredients in cosmetic products.

## Introduction

The proper functioning of the skin, the largest human organ largely responsible for homeostasis, is not an easy task. The skin is a physicochemical barrier that prevents microorganisms from the external environment from penetrating into deeper tissues. On the other hand, it is inhabited by microorganisms constituting its natural microflora. Like other microbiomes in the human body that are constantly inhabited by various microorganisms, the skin must show a certain level of tolerance to selected antigenic epitopes of microorganisms. The balance, characteristic of healthy skin, between immune tolerance and readiness for inflammation is established during the development of the skin microbiome and depends both on the state of the body’s immune system and on the qualitative and quantitative composition of the microbiome^1^. Therefore new solutions are sought to support the natural microbiome inhabiting the human skin. Natural cosmetic raw materials, including plant extracts present in cosmetic products, are known for their antioxidant, anti-aging, anti-inflammatory and whitening properties. In order to support the beneficial microorganisms inhabiting the human skin, it is worth thinking about products that, after applying to the skin, could also exhibit properties identical to plant extracts, so readily used in cosmetic products.

One of the methods used for the production of biologically active compounds, primarily in the food industry, but recently more and more frequently used in cosmetics, is fermentation, which may improve the quality of product and facilitate the absorption of active substances by the human body^2^. Due to its probiotic properties, scientists are increasingly paying attention to Kombucha, which is a popular drink among traditional fermented foods. Kombucha commonly known as a SCOBY is usually prepared by aerobic and static fermentation of a sucrose-sweetened medium, usually black tea with a symbiotic culture of acetic acid bacteria (AAB), lactic acid bacteria (LAB) and yeast^3–6^. Its fermentation process also leads to the formation of a floating biofilm on the surface of the growth medium due to the activity of certain strains of AAB^7^. Chemical analyses of Kombucha have shown the presence of various organic acids, such as acetic, gluconic, glucuronic, malic, L-lactic, malonic, oxalic and tartaric that are responsible for sour taste^8^; the vitamins: B_1_, B_2_, B_6_, B_12_, C^9,10^; minerals: Cu, Fe, Mn, Ni, Zn^9^; anions: F^−^, Cl^−^, Br^−^, I^−^, NO_3_^−^, HPO_4_^−^, SO_4_^−^^11^ and polyphenolic compounds^12^. That is why Kombucha, thanks to its rich content of active substances and probiotic properties, has a beneficial effect on health. Nonhuman research has demonstrated the antibacterial, antioxidant, hepatoprotective, antidiabetic, anti-inflammatory and anticancer properties of Kombucha tea^13^. Traditional substrate for the SCOBY fermentation is black or green tea, but scientific research have shown alternative substrates such as: Jerusalem artichoke extract, milk, fresh sweet whey, Coca-Cola, red and white wine*, Echinacea, Mentha*, cherry and grape juice, and more^13^. What is more, studies have shown that the composition and metabolite concentration depends on the inoculum source, the sugar and tea concentration, the fermentation time and the temperature used^14^.

Taking into consideration the opportunity of using substrates for fermentation other than tea, the possibility of using Yerba Mate as a fermentation medium was determined. Yerba Mate is a popular tea beverage produced and consumed in South American countries and is processed from leaves and stems of *Ilex paraguariensis*. Numerous active phytochemicals have been identified in Mate tea that may be responsible for its health benefits. Among them, there are polyphenolic acids such as chlorogenic acid, caffeic acid, 3, 4-dicaffeoylquinic acid, 3, 5-dicaffeoylquinic acid, as well as xanthines (caffeine and theobromine), flavonoids (quercetin, kaempferol, and rutin), amino acids, minerals (P, Fe, and Ca), and vitamins (C, B_1_, and B_2_)^15,16^. Due to the presence of these compounds, Mate tea shows antioxidant, antimicrobial, anticancer, antidiabetic properties^17–19^. Moreover, the presence of saponins, which in addition to being responsible for the expressive, bitter taste of Mate tea, also have anti-inflammatory properties^20^. Due to the high content of biologically active compounds, this infusion is often compared to those of black or green tea, therefore it seems to be a favorable medium for Kombucha fermentation.

The aim of the study was to evaluate the properties of ferments obtained from Yerba Mate extract after fermentation using cooperation in complex multi-species systems and investigate the impact of fermentation time on the biological activity of the obtained products. For this purpose, the content of various biologically active compounds of prepared ferments was evaluated using a high performance liquid chromatography. Cytotoxicity assessments were carried out on keratinocytes, and fibroblasts, using Alamar Blue and Neutral Red. The antioxidant potential of obtained ferments was analyzed by evaluating the scavenging of external (DPPH and ABTS method) and intracellular free radicals. The possibility of inhibiting the activity of the collagenase and elastase, which play an important role in the skin aging process through the degradation of collagen and elastin fibers were measured. In order to assess the anti-inflammatory properties of the studied ferments, the possibility of inhibiting lipoxygenase activity and the effect on protein denaturation were determined. In the final stage, the influence of the obtained ferments on skin moisture and transepidermal water loss was also evaluated.

## Results and discussion

### Determination of bioactive compounds

Plants have been used as a source of bioactive components for thousands of years. However, the need for more natural ingredients has increased over the past decade. Polyphenolic compounds derived from plant sources are widely found in cosmetic and pharmaceutical products and have been shown to have significant antioxidant effects^21^.

**Figure 1:**
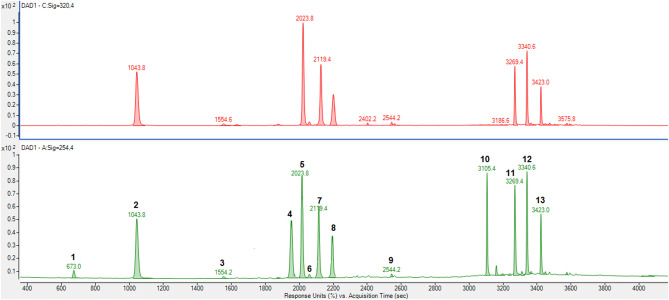
Example extracted ion chromatograms (XIC) obtained for Yerba Mate extract. Names of compounds indicated by numbers (1–13) explained in Table 1.

The composition of fermented and unfermented yerba extracts were analyzed using HPLC/ESI–MS. Bioactive compounds including phenolic acids, xanthines and flavonoids were identified. The detailed mass data are summarized in Table 1. The obtained profile was similar to those reported in literature^15,22,23^ and predominant components were isomers of caffeoylquinic acid (CQA) and dicaffeoylquinic acid (diCQA). The tested samples also contained a fair amount of flavonoid—rutin and caffeine belonging to a class of purine alkaloids—xanthines. The example of chromatogram Yerba Mate extract is presented in Fig. 1. Among identified compounds 10 were quantitatively analysed; caffeoyl-glucoside, 3- and 5-feruloylquinic acid were not quantified because their concentrations were very low. The obtained results expressed in mg/g of extract dry weight are presented in Table 2.

**Table 1:** Bioactive compounds detected using HPLC/ESI–MS.

Peak no	Ionisation	Theoretical ion mass	Observed ion mass (m/z)	Compound
1	Pos.	181.0720	181.0718	Theobromine
2	Neg.	353.0878	353.0887	3-Caffeoylquinic acid
3	Neg.	341.0878	341.0887	Caffeoyl-glucoside
4	Pos.	195.0877	195.0871	Caffeine
5	Neg.	353.0878	353.0876	5-Caffeoylquinic acid
6	Neg.	367.1035	367.1038	3-Feruloylquinic acid
7	Neg.	353.0878	353.0872	4-Caffeoylquinic acid
8	Neg.	179.0349	179.0350	Caffeic acid
9	Neg.	367.1035	367.1034	5-Feruloylquinic acid
10	Neg.	609.1461	609.1465	Rutin
11	Neg.	515.1195	515.1179	3,4-Dicaffeoylquinic acid
12	Neg.	515.1195	515.1185	3,5-Dicaffeoylquinic acid
13	Neg.	515.1195	515.1190	4,5-Dicaffeoylquinic acid

**Table 2:** Quantification results obtained for Yerba Mate extract end Kombucha ferments.

Compound	Content (mg/g of dry weight)				
	YM	F7	F14	F21	F35
Caffeic acid	0.81 ± 0.03	0.07 ± 0.01	0.09 ± 0.01	0.01 ± 0.01	0.06 ± 0.01
3-Caffeoylquinic acid	6.97 ± 0.24	8.45 ± 0.31	9.60 ± 0.42	15.54 ± 0.97	5.45 ± 0.32
4-Caffeoylquinic acid	3.85 ± 0.10	2.50 ± 0.08	4.82 ± 0.03	5.17 ± 0.11	2.54 ± 0.12
5-Caffeoylquinic acid	3.55 ± 0.11	4.06 ± 0.13	6.06 ± 0.12	7.79 ± 0.37	3.00 ± 0.14
3,4-Dicaffeoylquinic acid	1.14 ± 0.06	0.86 ± 0.02	1.54 ± 0.09	1.41 ± 0.02	0.79 ± 0.02
3,5-Dicaffeoylquinic acid	1.91 ± 0.04	3.38 ± 0.04	3.06 ± 0.15	4.89 ± 0.02	1.54 ± 0.06
4,5-Dicaffeoylquinic acid	1.36 ± 0.07	1.42 ± 0.02	2.08 ± 0.07	2.16 ± 0.09	1.03 ± 0.06
Sum of quantified phenolic acids	19.59	20.74	27.25	36.97	14.41
Theobromine	0.26 ± 0.02	0.24 ± 0.01	0.31 ± 0.01	0.43 ± 0.01	0.17 ± 0.01
Caffeine	2.84 ± 0.11	2.28 ± 0.05	2.47 ± 0.08	3.83 ± 0.04	1.66 ± 0.01
Rutin	1.14 ± 0.06	1.34 ± 0.09	1.71 ± 0.07	2.47 ± 0.03	1.03 ± 0.06
Sum of quantified compounds	23.83	24.60	31.74	43.70	17.27

Values are means ± standard deviation (SD) of triplicate.

As can be seen, the content of determined compounds varied among the analyzed extracts. The total content of phenolic compounds in the tested samples was the highest for the ferment after 21 days of fermentation (F21) compared to the Yerba Mate extract. F14 sample was also rich in the tested phenolic compounds. Moreover, extending the fermentation time to 35 days, a significant decrease in the content of polyphenols was visible. It was found that the 3-CQA was the most abundant component (even 15.54 ± 0.97 mg/g for F21 sample and 6,97 ± 0.24 mg/g for YM extract). The concentration of caffeic acid was the lowest in all the samples analyzed. Regarding the analysis of methylxanthines, among the plant samples examined, the highest caffeine and theobromine contents were also found for F21 extract (0.43 ± 0.01 mg/g theobromine and 3.83 ± 0.04 mg/g caffeine) compared to other extracts (the lowest content of alkaloids for the F35 sample). The low content of theobromine compared to caffeine may be due to caffeine being synthesized from the xanthosine, via 7-methylxanthosine, 7-methylxanthine and theobromine. Theobromine is methylated to caffeine which would be the final metabolite^24^. In addition, the flavonoid was analyzed. The results showed the highest rutin content for the F21 sample and the lowest for F35. These results correspond to the total content of bioactive compounds.

In summary, the ferments obtained from the Yerba Mate extract and Kombucha showed a high content of active compounds compared to the Mate extract. Note that many authors have shown that YM extract represents an important source of polyphenols, alkaloids and flavonoids with a high antioxidative capacity^17,25^. The antioxidant activity of Kombucha is due to its presence, among others, of polyphenols. Kombucha ferments had higher antioxidant activity than Yerba Mate extract, and this may be due to the production of low-molecular-weight ingredients and structural modifications of tea polyphenols by enzymes produced by bacteria and yeast during fermentation^26^. Moreover, Chakravorty et al. evaluated the polyphenol content and the antioxidant activity of Kombucha tea during the course of its fermentation (0, 7, 14, and 21 days) and observed a high tendency to increase especially after the 7th days, which may be due to the higher microbial diversity achieved by that time^27^.

### Assessment of antioxidant activity

Plant raw materials with strong antioxidant properties, thanks to which they play a key role to our body. Their action is mainly based on the elimination of reactive oxygen and nitrogen species, neutralization of free radicals, chelation of iron and copper ions of metals and inhibition of enzymes from the group of oxidases^14,28–30^. A valuable source of secondary metabolites are both extracts and ferments obtained from the Yerba Mate. As a result, seven measurement points were realized for each concentration (10 µg/mL, 100 µg/mL, 250 µg/mL, 500 µg/mL, 1000 µg/mL) of the tested extract. From these values, the IC50 point was determined for the data obtained after 30 min (Table 3). It was shown that the lowest values of 175.93 ± 0.19 µg/mL were shown by the ferment obtained after 21 days of fermentation of Mate tea with Kombucha. Compared to YM extract (IC_50_ = 263.37 ± 0.17 µg/mL). This value was 33% lower than the value obtained for the Mate extract without the fermentation process. F21 showed a higher antioxidant capacity. Similar conclusions regarding time of fermentation were reached by Floegel et al., because by extending the fermentation time, the antioxidant potential of the raw material was also increasing along with the longer fermentation time of tea^31,32^.

**Table 3:** Values of IC50 of DPPH and ABTS for Yerba Mate extract and ferments after 30 min of exposure (for DPPH method).

Sample	YM	F7	F14	F21	F35
**DPPH radical scavenging assay**					
IC_50_ (µg/mL)	263.37 ± 0.17	219.04 ± 0.11	221.84 ± 0.20	175.93 ± 0.19	292.85 ± 0.23
**ABTS + scavenging assay**					
IC_50_ (µg/mL)	160.85 ± 0.07	172.58 ± 0.02	174.02 ± 0.14	158.85 ± 0.08	182.68 ± 0.12

Values are means ± standard deviation (SD) of triplicate.

**Figure 2:**
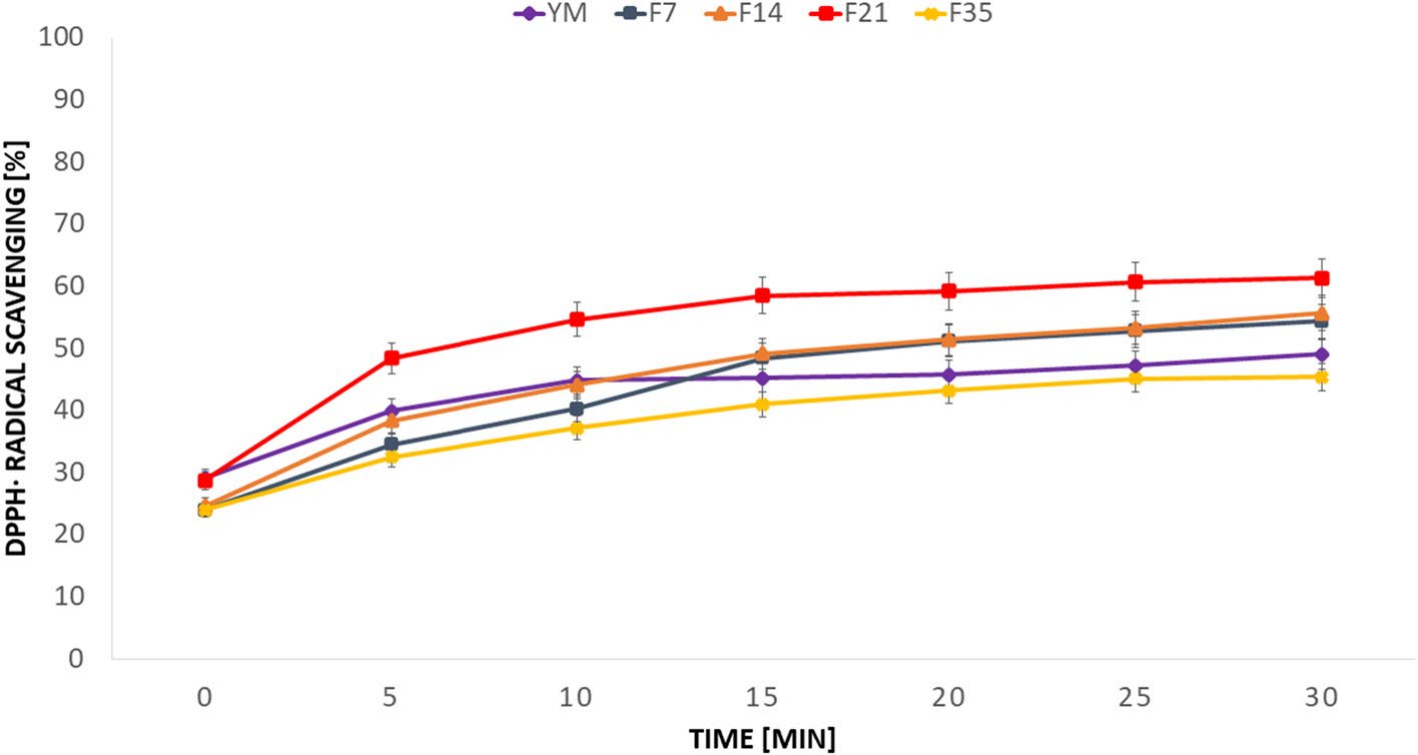
The changes DPPH radical scavenging capacity over time for 250 μg/mL of Yerba Mate extract and Kombucha ferments. Values are mean of three replicate determinations (n = 3).

The diagram presented in Fig. 2 referring to the DPPH radical scavenging capacity for the extract and Yerba Mate ferment shows the data for the concentration of 250 µg/mL. The obtained data showed that the analyzed extracts show a variety of antioxidant properties. For the discussed concentration, for all the time-being of the experiment the lowest values were observed in the case of F35 extract and the highest antioxidant properties were exhibited by F21 extract, while values for the rest of extracts were noted between values of those. At the end of the experiment, meaning after 30 min, scavenging capacity of F35 was 44.8% and 61.7% for the F21. An interesting behavior was observed for the pure YM extract, where after the time of 10 min scavenging capacity growth has slowed down compared to the rest of analysed extracts.

The ABTS radical neutralization analysis was also performed for the concentrations of 10 µg/mL, 100 µg/mL, 250 µg/mL and 1000 µg/mL. Based on the obtained values, the IC50 point was determined (Table 3). Analogously to the previously discussed DPPH radical, the most favourable values were observed for the ferments obtained after 21 days. Value of 158.85 µg/mL obtained in this case was lowest by around 2% compared to the Yerba Mate extract not subjected to the fermentation process, where IC50 was 160.85 µg/mL. Lower values of IC50 for extracts subjected to the fermentation process with Kombucha may be caused by bacteria and yeast released in the fermentation process, which may result in better efficiency on nitrogen and superoxide radicals, and poor scavenging performance on hydroxyl radicals^12^.

**Figure 3:**
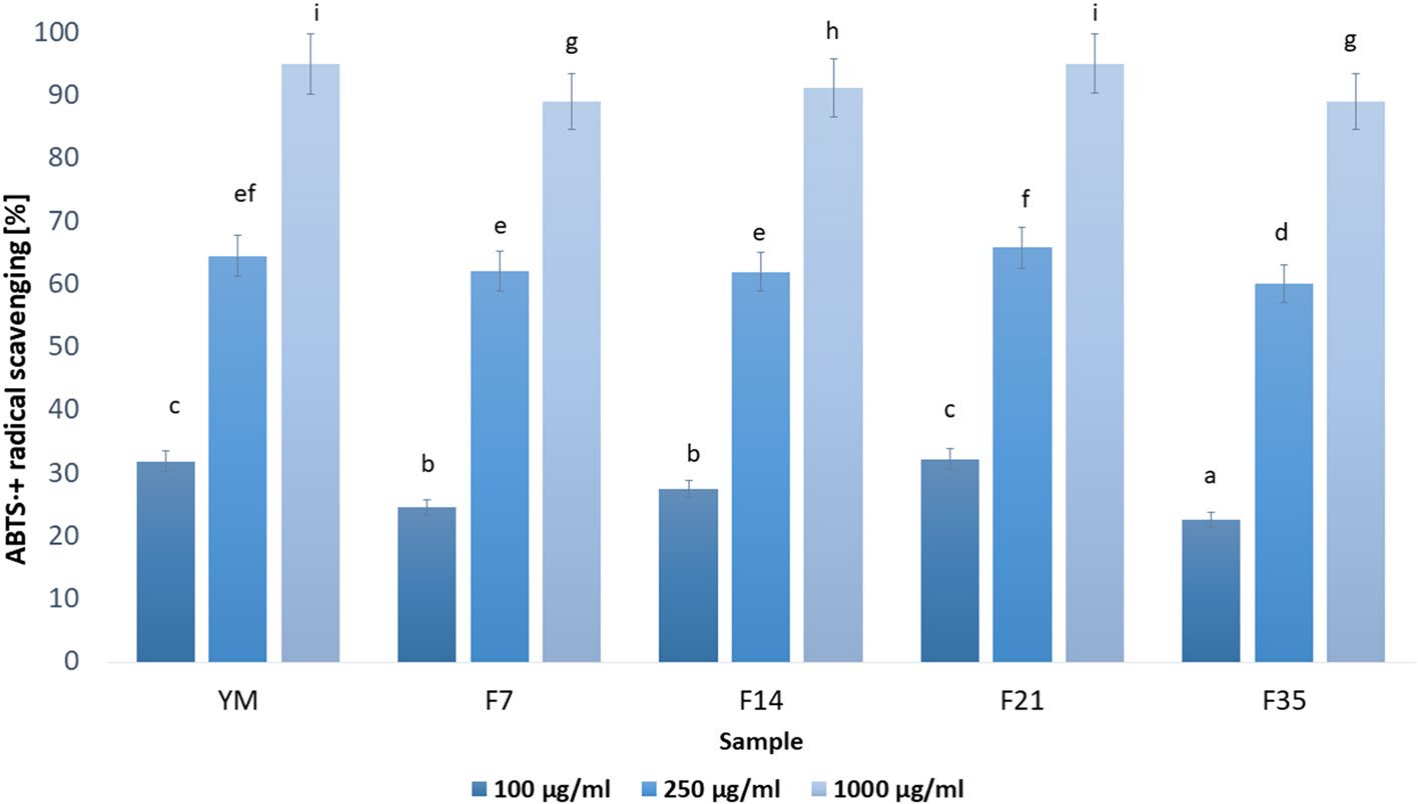
ABTS + radical scavenging by various concentrations of Yerba Mate extracts and Kombucha ferments. Values are means of three replicate determinations (n = 3). Different letters on the charts indicate significant differences between groups (*p* < 0.05).

The diagram presented in Fig. 3 referring to the ABTS radical scavenging capacity for the extract and Yerba Mate ferment shows the data for the concentration of 100, 250 and 1000 µg/mL. A conclusion can be drawn that the higher concentration of the extract the higher free radical scavenging ability, in each discussed case. Despite the significant reduction of free radicals for all the samples tested (about 90% at 1000 µg/mL), the highest capacity of the extract and ferments is observed for YM and F21 (above 90% ABTS + radical scavenging).

In the next stage of the research, the ability of the YM extract and the obtained ferments to reduce intracellular production of reactive oxygen species in two skin cell lines—fibroblasts (BJ) and keratinocytes (HaCaT) was assessed. Reactive oxygen species (ROS) produced internally and extracellularly play a key role in cellular physiopathology^33^. They influence, inter alia, cell proliferation and a number of different signaling pathways^34^. The excess of free radicals in cells can damage proteins, lipids and DNA, therefore it is extremely important to maintain redox homeostasis, which is closely related to the ratio of the amount of oxidants and antioxidants. This study was performed using fluorogenic H2DCFDA dye. After passive diffusion of H2DCFDA into HaCaT and BJ cells, it is deacetylated by intracellular esterases to a non‐fluorescent compound. In the presence of ROS, it is oxidized into highly fluorescent 2′,7′‐dichlorofluorescein (DCF). H2DCFDA probe was used to detect redox imbalance after exposure of the test cells to YM extract and ferments, as it can react with several ROS, including hydroxyl radicals, hydrogen peroxide and peroxynitrite^35^.

**Figure 4:**
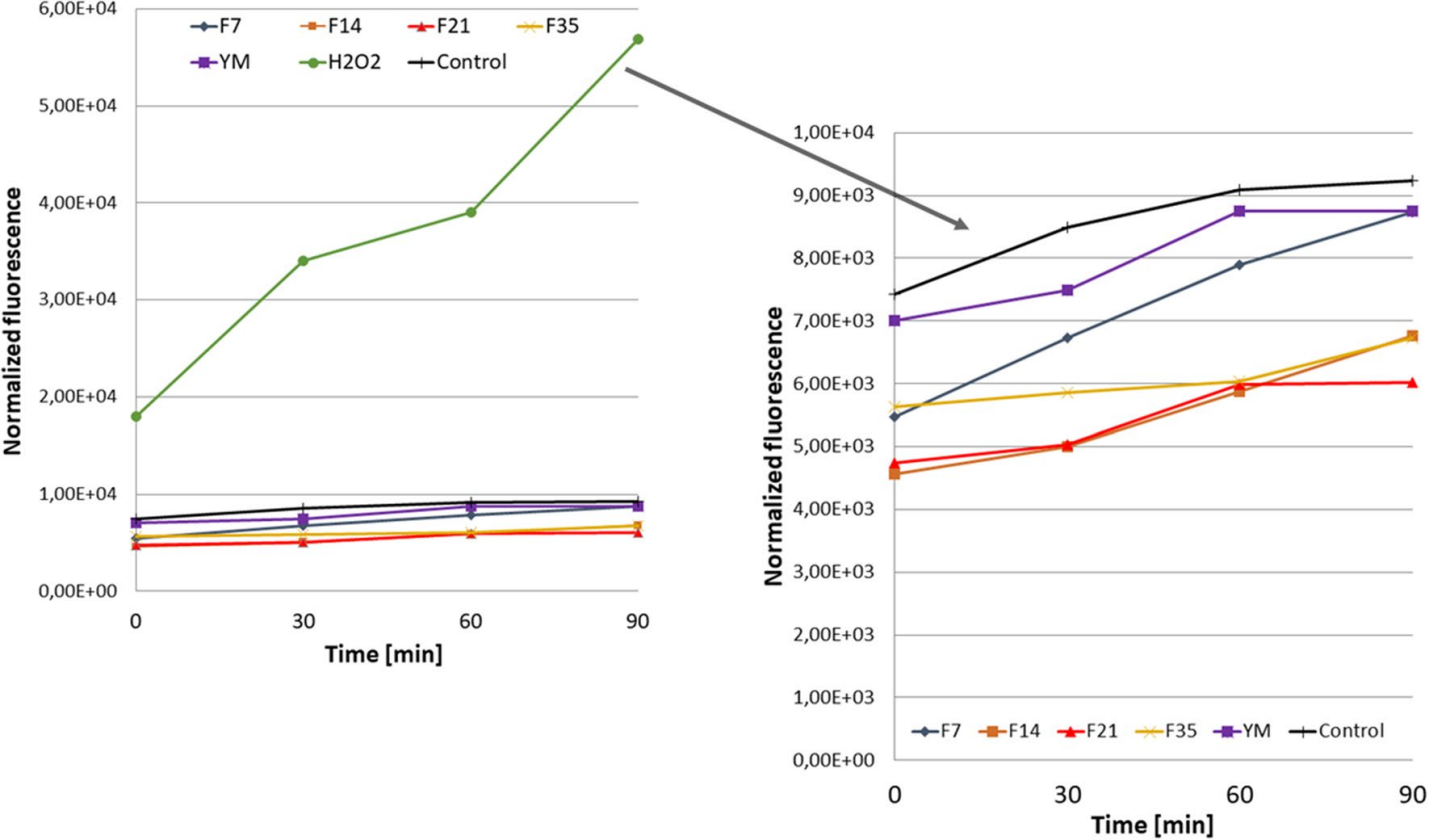
Effect of Yerba Mate and Kombucha ferments (at a concentration of 250 µg/mL) on the DCF fluorescence in fibroblasts. Data are expressed as the mean of 3 independent experiments each consisting of three replicates per treatment group.

**Figure 5:**
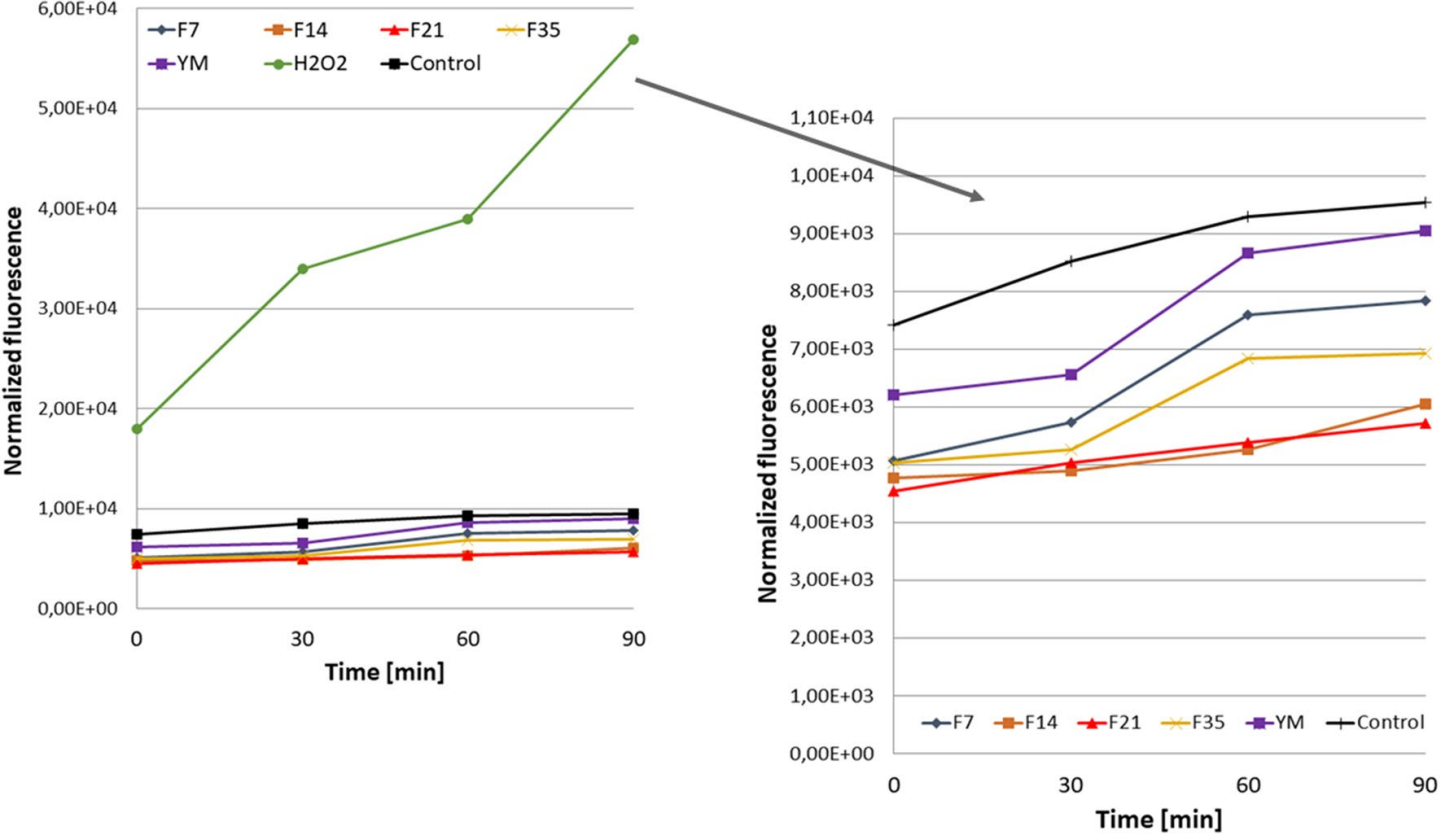
Effect of Yerba Mate and Kombucha ferments (at a concentration of 250 µg/mL) on the DCF fluorescence in HaCaT cells. Data are expressed as the mean of 3 independent experiments each consisting of three replicates per treatment group.

The analysis carried out as part of this study demonstrated that both the YM extract and the obtained ferments can reduce the intracellular level of free radicals in both fibroblasts and keratinocytes (HaCaT), because the values of normalized fluorescence were lower than in control cells (cells grown in the medium without addition extract or ferments) (Figs. 4, 5). As part of the research, the effect of the extract and ferments in the range of 10–1000 µg/mL was examined, while the figures show the results for the concentration of 250 µm/mL. Th significantly reduce the level of free radicals for fibroblasts and keratinocytes have showed for ferments after 14 and 21 days of fermentation with Kombucha, but all tested samples are capable of reducing this level. Similar results were obtained for the remaining tested concentrations of the extract and ferments (data not shown). The least favorable results were obtained for the ferments after 35 days, which may indicate that too long fermentation time may contribute to the accumulation of harmful fermentation products that may affect formation of free radicals inside cells^12,36^.

### Cytotoxicity assessment

In order to assess the potential cytotoxicity of the tested extract and the ferments, the Alamar Blue and Neutral Red tests were used. The performance of these tests allowed the assessment of the influence of the tested samples on the metabolic activity and proliferation of fibroblasts and keratinocytes. The first of the tests used is based on the application of non-fluorescent resazurin, which can be reduced to fluorescent resorufin. This test allows the assessment of cell viability, their proliferation and the determination of mitochondrial respiratory activity^37^. In contrast, the neutral reduptake test relies on the ability of living cells to incorporate and bind the neutral red dye in lysosomes. This uptake is strictly dependent on the ability of cells to maintain pH gradients, which is closely related to the production of adenosine triphosphate (ATP)^38^.

**Figure 6:**
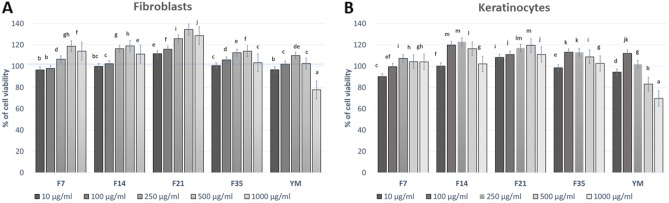
Effect of the increase in the concentration of extract and ferments (10–1000 μg/mL) on cell viability (Neutral Red assay) by cultured fibroblasts (**A**) and keratinocytes (**B**) after 24 h exposure. Data are the mean ± SD of three independent experiments each consisting of three replicates per test group. Different letters on the charts indicate significant differences between groups (*p* < 0.05).

**Figure 7:**
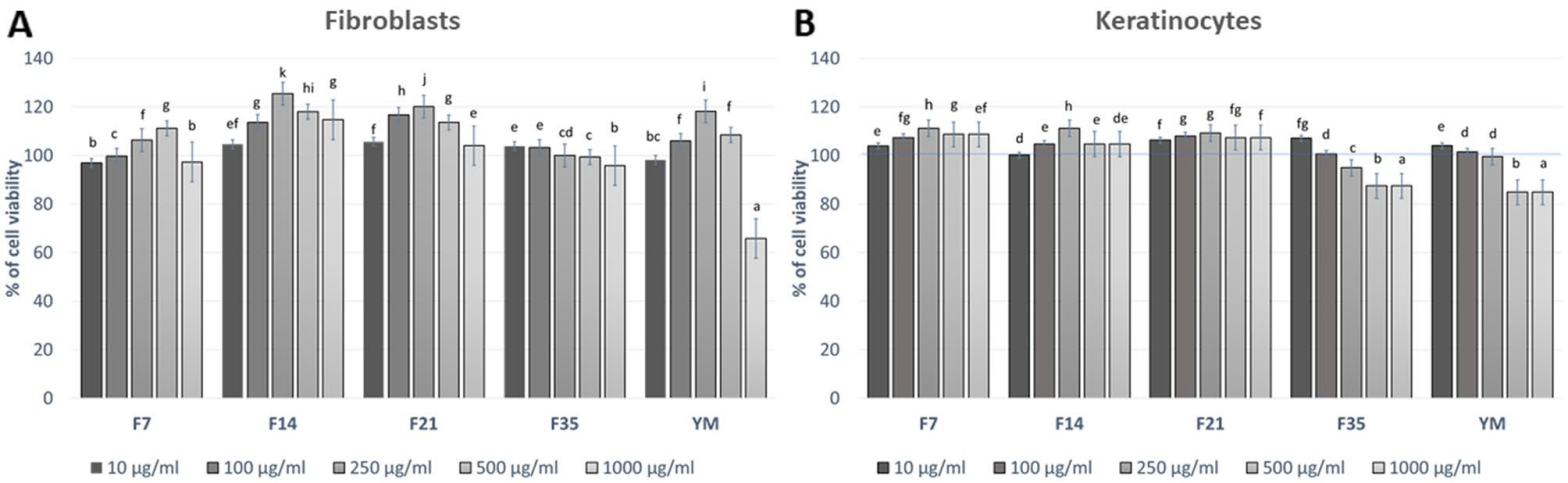
Effect of the increase in the concentration of extract and ferments (10–1000 μg/mL) on cell viability (Alamar Blue assay) by cultured fibroblasts (**A**) and keratinocytes (**B**). Data are the mean ± SD of three independent experiments each consisting of three replicates of each test concentration. Different letters on the charts indicate significant differences between groups (*p* < 0.05).

The cytotoxicity analysis carried out as part of this study showed that the effect of the studied extract and Kombucha ferments on the viability of skin cells in vitro is dependent both on the dose used and the fermentation time of Yerba Mate. The results of the Neutral Red test indicated that the obtained ferments may have a positive effect on the viability of both fibroblasts and keratinocytes (Fig. 6). The significantly highest cell viability of BJ (almost 130%) have shown when treated with 500 µg/mL of F21. Similas results have determined for HaCaT—almost 120% cell of viability after treated with 500 µg/mL of F14 and F21. Additionally, the analyzes carried out showed that the YM extract at the highest concentration used (1000 µg/mL) caused a cytotoxic effect on fibroblasts. In the case of HaCaT cells, this effect was observed for the two highest tested concentrations (500 and 1000 µg/mL), which indicates that the obtained ferments have a more beneficial effect on skin cells than the extract. Similar results were obtained using the test with resazurin, which also indicated the possibility of cytoprotective effect of the obtained ferments on the viability of the examined cells. For fibloblasts—almost 120% cell of viability after treated with 250 µg/mL of F14 and F21 (Fig. 7). This test showed that the tested ferments are able to increase the viability of fibroblasts to a greater extent than keratinocytes. As in the previous test, the most noticeable increase in the metabolic activity of fibroblasts was observed in the F14 and F21 ferment, while the F35 ferment showed no positive effect on the viability of these cells. In the case of HaCaT cells, a much smaller increase in viability and a cytotoxic effect were observed in the case of higher concentrations of F35 ferment and YM extract. The obtained results indicate that the compounds formed during the fermentation of Mate tea with Kombucha may positively affect the viability of skin cells, which indicates their potential use in various preparations used for skin care. There are also recent scientific reports on the properties of Yerba Mate ferments obtained with Kombucha, but their influence on the viability of skin cells has not been described so far. The cytoprotective effect of the analyzed ferments on skin cells shown in this work is undoubtedly associated with a wide range of biologically active compounds, the presence of which has been confirmed in chromatographic analysis. These compounds can protect cells by reducing oxidative stress, increase metabolic activity of cells and counteract adverse changes in cell structure^39–43^.

### Assessment of matrix metallopeptidades inhibition

Skin aging is a biological phenomenon that is influenced by many internal and external factors. Due to the fact that the skin is an organ directly exposed to the environment, it has to cope with unfavorable UV radiation, which generates an increase in reactive oxygen species. ROS are able to initiate complex molecular pathways, including activation of enzymes that degrade extracellular matrix proteins (ECM) in the dermis, such as collagen and elastin. Activation of the protease results in the breakdown, fragmentation and disorganization of ECM proteins, visibly manifested by typical UV-induced skin changes, such as deep wrinkles, loss of color and elasticity of the skin^44–46^. Matrix metalloproteinases (MMPs), a family of zinc-containing multidomain endopeptidases with a broad range of substrate specificity, are the major group of proteolytic enzymes involved in the degradation of skin connective tissue components^47^. Inhibition of the activity of ECM-degrading proteins, such as collagenase and elastase, may be a useful approach to prevent UV-induced skin lesions and premature skin aging^48^. Therefore, in order to assess the potential use of the studied extracts in the fight against skin aging, the possibility of Yerba Mate extract and Kombucha ferments obtained from YM extract for inhibiting the activity of the collagenase and elastase enzymes were investigated.

**Figure 8:**
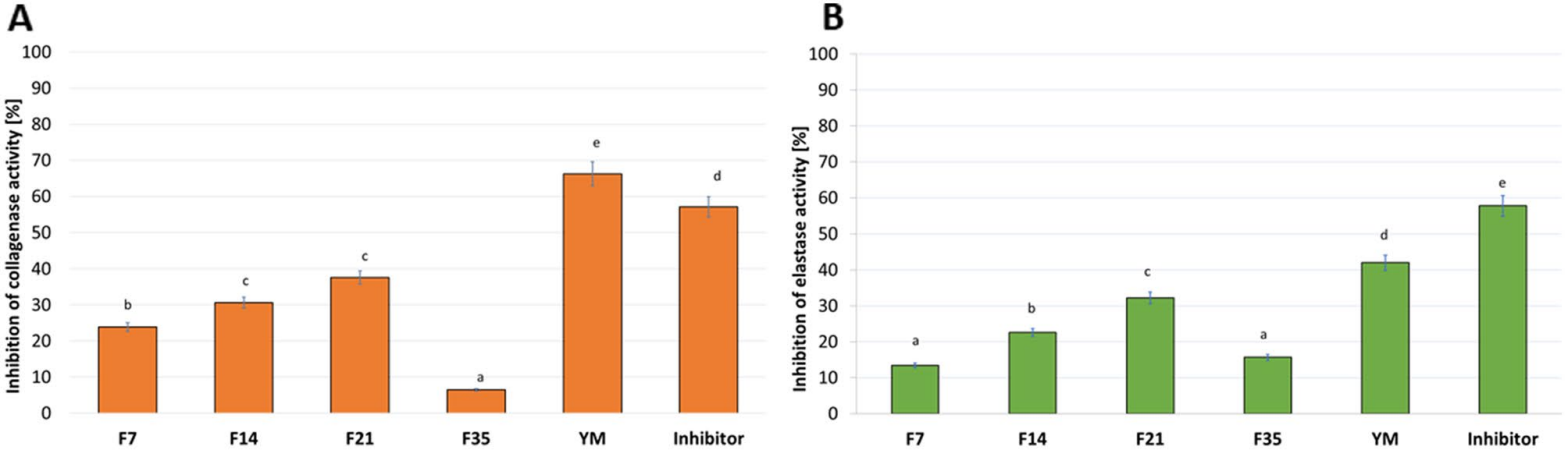
Influence of Yerba Mate extract and Kombucha ferments on collagenase (**A**) and elastase (**B**) inhibition. Data are the mean ± SD of three independent experiments, each of which consists of three replicates per treatment group. Different letters on the charts indicate significant differences between groups (*p* < 0.05).

The conducted analysis showed that the tested samples differ in their ability to inhibit collagenase and elastase depending on the fermentation time of Kombucha, which results in the inhibition of substrate hydrolysis. The analyzes of the extract and ferments were carried out for the concentration of 500 µg/mL. It was observed that the significantly highest (approximately 65%, *p* < 0.05) anti-collagenase activity had Yerba Mate extract (Fig. 8). Regarding Kombucha ferments, lower anti-collagenase activity was observed compared to Mate tea extract. However, by extending the fermentation time to 21 days, an increase in the ability to inhibit collagenase activity was visible (almost 40% for F21 sample). After this time, this activity decreased (for F35 sample only 5% enzyme inhibition capacity). Similar results have been observed for the anti-elastase activity presented also in Fig. 8. The YM extract had the significantly highest ability to inhibit elastase at a level above 40%, while the ferment after 21 days of fermentation also showed significantly second-highest anti-elastase activity at the level of 32%.

Phenolic extracts are found to inhibit the activity of proteinases, which catalyze the degradation of skin proteins, such as collagen and elastin. Collagen in the dermis is responsible for the firmness, elastin fibers lend the elasticity^48,49^. Thring et al. determined anti-collagenase, anti-elastase and antioxidant activities of 21 plant extracts and correlated them with the total phenolic content. The white tea extract showed the highest inhibitory activity against both enzymes as well as the highest antioxidant activity and phenolic content^50^. The presence of active ingredients such as phenolic compounds, methylxanthines and flavonoids in Yerba Mate extract may help to inhibit the activity of enzymes responsible for skin aging^17^. In addition, more and more research concerns fermented plant extracts, which are a rich source of antioxidants, vitamins, minerals, polyphenols as well as probiotics^51^. Kim et al. have shown that aqueous extract of *Fructus arctii* (FFAE) fermented with *Grifola frondosa* HB0071 exhibited 5-lipoxygenase inhibitory and antioxidant activities. FFAE inhibits the expression of matrix metalloproteinase (MMP-1) in UV-A treated human fibroblasts in a dose-dependent manner^52^. Moreover, *Saccharomyces cerevisiae* mediated fermented black ginseng (FBG) has been reported for the anti-wrinkle activity in cultured human fibroblasts HS68 and proved that FBG was noncytotoxic. FBG treatment increased the expression of type I procollagen and tissue inhibitor of metalloproteinase-2 and reduced the expression of MMP-1, MMP-2, and MMP-9 in fibroblasts cells^53^.

### Assessment of anti-inflammatory potential

**Figure 9:**
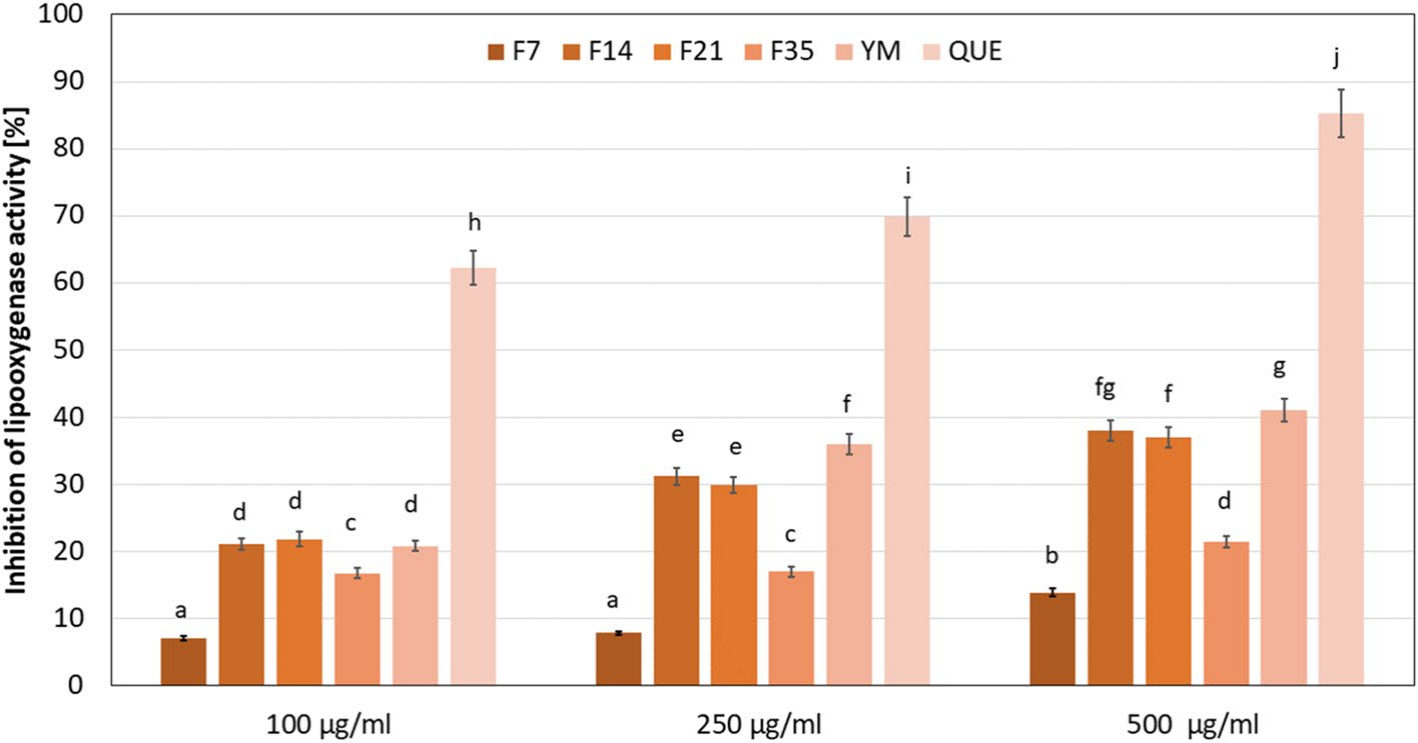
Anti-inflammatory effect of Yerba Mate extract and ferments. Data are the mean ± SD of three independent experiments, each of which consists of three replicates per treatment group. Different letters on the charts indicate significant differences between groups (*p* < 0.05). *QUE* quercetin.

The active substances contained in some plant extracts may also have anti-inflammatory effects. As shown Oguntibeju^54^, the anti-inflammatory properties of plant extracts can be based on various mechanisms of action, which depend on the type of active compounds. As the most important mechanisms are proposed inhibition of 15-lipoxygenase (LOX), the key enzyme in the synthesis of pro-inflammatory reactions mediators—leukotrienes, inhibition of prostaglandins synthesis (COX), inhibition of cytokines with pro-inflammatory effects, and inhibition of phospholipase A2, what contributes to limiting of LOX and COX activity and plays the major role in the inflammation treatment^54–58^. In this research, the anti-inflammatory properties of the Yerba ferments and extract were performed. Determination of the LOX inhibition was used. The results are presented in Fig. 9.

Based on the obtained results, it was observed that both the Yerba ferments and the extract showed the moderate ability to LOX inhibition. The anti-inflammatory properties of the analyzed samples depended on their concentration and the time of the fermentation process. In the range of analyzed concentrations, the strongest ability to inhibit LOX activity was observed for the F14 and F21 and the extract samples. At the concentration of 100 µg/mL, the differences were not statistically significant and the analyzed parameter was about 20%. With the increased concentration of the F14 and F21 ferments and the extract, it was noted a two-time increase in the anti-inflammatory properties, and the value of analyzed parameter was about 30–35% at the concentration of 250 µg/mL and 35–40% at the concentration of 500 µg/mL (significantly higher values were observed for the YM extract). The F7 and F35 samples were characterized by lower anti-inflammatory effects. For the F7 and the F35 ferments, respectively, about 60% and about 45% lower LOX inhibition was shown in relation to the F14 and F21 samples.

Previous studies indicated that active compounds contained in Yerba Mate extracts have a strong ability to inhibit pro-inflammatory factors. In vitro studies of Puangpraphant et al.^58^ have shown that quercetin and saponins derived from the aqueous YM extract are the main ingredients responsible for its anti-inflammatory effect and these substances are capable of decreasing the production of interleukin-6 (IL-6), cyclooxygenases (COX-2) and nitric oxide (NO), the main mediators of the inflammatory process. Heck et al.^59^ have also shown that tannins contained in Mate tea extracts contribute to a decrease in LOX activity. Anti-inflammatory properties of YM, through reduction of COX-2 and nitric oxide synthase, were indicated by Guollermo et al.^60^ in the in vivo models.

### Transepidermal water loss (TEWL) and skin hydratation measurements

**Figure 10:**
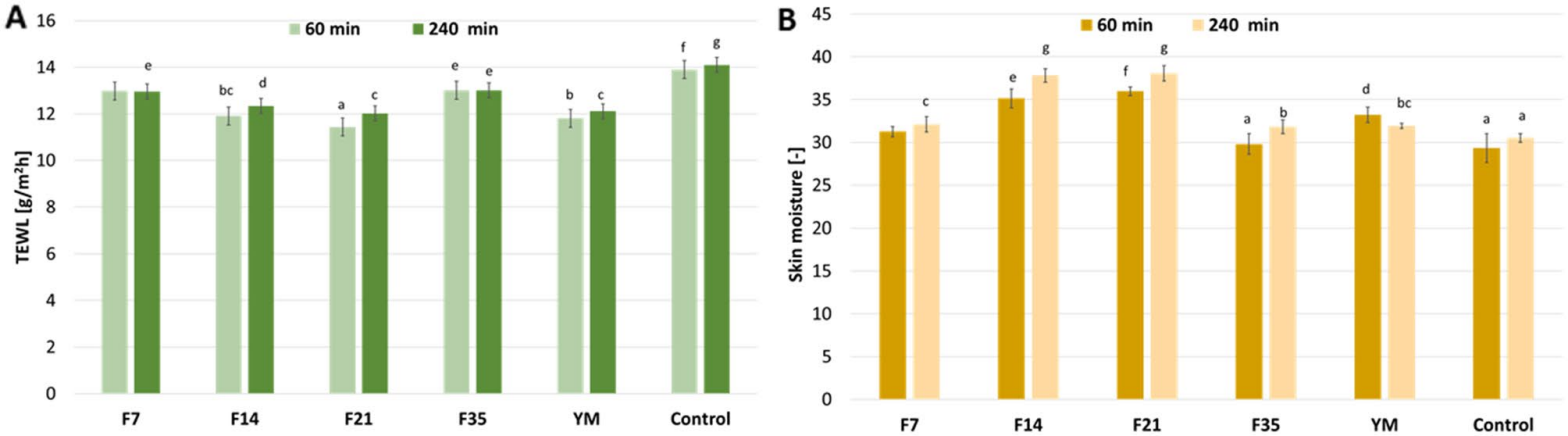
Influence of Yerba Mate extract and ferments (100 µg/mL) on transepidermal water loss (TEWL) (**A**) and skin hydration (**B**). The determinations were made in five replicates. Different letters on the charts indicate significant differences between groups (*p* < 0.05).

As a rich source of various active substances, plant extracts may have beneficial effects not only as a result of internal administration but also when they are applied to the skin surface. This property of plant extracts is used in the cosmetic and pharmaceutical industry, where plant extracts are often used as active ingredients of the products like creams, balms and ointment. Applied to the skin, plant extracts may contribute to an increase in skin moisture and prevent excessive loss of water from the epidermis. Proper skin hydration plays an important role in many processes, which most expected are acceleration of wound healing and skin regeneration and delaying skin aging processes. The regeneration of dry and deprived of adequate moisture level skin is more difficult. Lack of suitable moisture in the skin may also significantly inhibit the healing of wounds and cause its bad condition. Because of that, the impact of the analyzed ferments and the extract on the skin moisture and transepidermal water loss (TEWL) was carried out. The study was conducted with samples in a concentration of 100 µg/mL. The results are presented in Fig. 10.

It was observed that the application of the Yerba ferments and extract to the skin improves the hydration level. An increase in hydration depended on the fermentation time of the extracts. Relative to the control field, where no sample was applied, the strongest ability to improve the skin hydration was noted for F14 and F21 ferments. In relation to the control field, an increase of about 20% and 30% was obtained after 60 and 240 min, respectively. The analyzed parameter was not significantly different for these samples. Lower values of skin hydration, about 5% higher than for the control and not significantly different from Yerba extract, were observed for the F7 ferment. The weakest skin moisturizing properties were noted for the ferment after 35 days of fermentation. The obtained results were about 30 and 31.5 skin moisture units (60 and 240 min respectively) and achieved a level not significantly different than for the control field. It was also observed that the ferments are characterized by long-lasting moisturizing properties. In contrast to the Yerba extract, for the analyzed ferments, there was observed an increase of the skin moisture level 240 min after their application to the skin. After the same time, for the extract, the skin moisture level decreased and returned to the physiological state.

The influence of the analyzed samples on a TEWL level was also carried out. The obtained results were analogous to the notes in the skin moisture assay. The most preferable TEWL lowering properties were observed for the F14 and F21 ferment samples and the Yerba extract. Relative to the control field, applying these samples to the skin surface reduced the TEWL value of about 15% and 10% after 60 and 240 min, respectively. The results obtained for the F7 and F35 ferments were about 5–7% lower than those for the control.

The cosmetic properties of plant extracts related to their influence on the skin condition, including a moisturizing ability, are because extracts contain a wide range of natural substances able to bind and retain water in the upper layers of the epidermis. Antioxidants (polyphenols, flavonoids), proteins and amino acids, and carbohydrates are indicated as the main compounds of plant extracts with moisturizing properties. These kinds of substances contain hydroxyl groups in their molecules that may bind water molecules by forming hydrogen bonds. Low molecular weight compounds included in plant extracts can penetrate deeper skin layers and are responsible for moisturizing the skin by keeping water in the skin, while the substances with a higher molecular weight and poorer skin penetration ability (e.g. carbohydrates and proteins) can act by occlusion on the skin surface limiting water evaporation from the epidermis. The reason for differences in the obtained results is probably a different composition of the analyzed samples, which depends on the fermentation time. Chu and Chen^12^ showed that changes in the concentration of bioactive substances in the fermentation process may be a result of the polymerization of simple compounds into the higher molecular weight compounds that occur in the first days of the fermentation process. In the next stage of fermentation, compounds may be depolymerized into simple substances, and in the final stage may be degraded. In the 14. and 21. days of the Yerba fermentation, the concentration of active ingredients is the highest, and due to that, moisturizing and the TEWL lowering properties are the most preferable for these ferments.

## Materials and methods

### Plant material and fermentation procedure

The analyses were conducted using natural processed leaves of Yerba Mate (*Ilex paraguaiensis* St. Hil.), obtained from a local and ecological factory (Alto Parana, Paraguay). The collection and storage of the material were carried out under strictly controlled conditions. Kombucha starter cultures were purchased from a commercial source from Poland. Before starting the fermentation process, Kombucha starter culture was stored under aseptic conditions in a refrigerator (4 °C) and consisted of acid broth and cellulose layer. Initially, an infusion of Mate tea was prepared in a sterile beaker by mixing 15 g of Yerba Mate with humidity of 8% (3% w/w), 50 g of sucrose (final concentration 10.0% m/v) and 500 mL of hot distilled water (95 °C). The mixture prepared in this way was stirred every few minutes with a glass rod until the solution cooled down (about 25 °C, cooling bath, cooling time 30–40 min). The resulting YM infusion was then filtered twice through membrane filters into sterile glass beakers (1000 mL, 18 cm height and 8 cm diameter). Then, tea fungus (3 g) and Kombucha (50 mL) were added to the filtrate and fermentation was carried out for 7, 14, 21 and 35 days (in separate beakers) at room temperature (about 25 °C). After fermentation, the obtained Kombucha was filtered and evaporated under reduced pressure at 40 °C. Ferments obtained after 7 days were signed as F7, after 14 days as F14, after 21 days as F21 and after 35 days as F35. Mate infusion without Kombucha was marked as YM.

### Determination of biologically active compounds

Main metabolites (chlorogenic acid, caffeic acid, 3, 4-dicaffeoylquinic acid, 3, 5-dicaffeoylquinic acid, caffeine, theobromine and rutin) were analysed according to the procedure described previously by Klejdus et al. and Oszmianski et al.^61,62^ using an ultra high performance liquid chromatography (UHPLC) Infinity Series II with DAD detector and Agilent 6224 ESI/TOF mass detector (Agilent Technologies, Santa Clara, CA). HPLC conditions were as follows: an RP18 reversed-phase column Titan (Supelco, Sigma-Aldrich) (10 cm × 2.1 mm i.d., 1.9 μm particle size), a thermostat temperature of 20 °C, and a flow rate of 0.2 mL/min. A mixture of acetonitrile with 0.05% of formic acid (solvent A) and water with 0.05% of formic acid (solvent B) were used as a mobile phase. The compounds were separated using gradient elution according to the program: 0–32 min from 10% A to 20% A, (from 90% B to 80% B), and 32–60 min from 20% A to 40% A (from 80% B to 60% B). Chromatograms were recorded from 200 to 400 nm. LC–MS analysis: the ion source operating parameters were as follow: drying gas temperature 325 °C, drying gas flow 5 l min^−1^, nebulizer pressure 30 psi, capillary voltage 3500 V, fragmentator 170 V, and skimmer 65 V. Ions were acquired in the range of 100 to 1050 m/z. MS identification was performed based on literature data and NIST database. Quantification was based on calibration curves obtained using methanol standard solutions of identified compounds. The content of the tested compounds is given in mg/g on a dry weight basis. All standards were from Sigma-Aldrich (St. Louis, MO, USA).

### Assessment of antioxidant activity

#### DPPH radical scavenging assay

The 1,1-diphenyl-2-picrylhydrazyl radical (DPPH) was used for determination of free radical-scavenging activity of the analyzed samples. This method was described by Brand-Williams et al.^63^. Initially, 33 µL of aqueous solutions of F7, F14, F21 and F35 ferments and YM extract at concentrations of 10 µg/mL to 1000 µg/mL were added to 167 µL methanol solution of DPPH (4 mM) in a 96-well plate. The mixture was shaken vigorously, and absorbance was measured at λ = 516 nm with FilterMax F5 microplate reader (Thermo Fisher Scientific, Waltham, MA, USA). The DPPH neutralization analysis was carried out for a period of 30 min, measurements were performed at 5-min intervals, in triplicate for each sample. As a control, distilled water mixed with DPPH solution was used. Measurements were carried out in triplicate for each extract sample. The scavenging activity on the DPPH radical was expressed as inhibition percentage using the following Eq. (1):

1 $$ \% {\text{ DPPH}}\;{\text{scavenging }} = \frac{Ac - As}{{Ac}} \times 100, $$

where: Ac is the absorbance of the control sample (containing DPPH and water), As is the absorbance of the test sample (containing DPPH and test sample).

#### ABTS + svavenging assay

Scavenging of ABTS·+ (2,20-Azino-bis(3-ethylbenzothiazoline-6-sulfonic acid) diammonium salt) free radical was evaluated according to the procedure described by Arnao et al.^64^. The stock solution included 7 mM ABTS solution and 2.4 mM potassium persulfate solution. The working solution was then prepared by mixing the two stock solutions in equal quantities and allowing them to react for 14 h at room temperature in the dark. The solution was then diluted by mixing 1 mL ABTS solution with 40 mL methanol to obtain an absorbance of 0.706 ± 0.01 units at 734 nm using a spectrophotometer. Then, F7, F14, F21, F35 ferments and YM extract (1 mL) were allowed to react with 1 mL of the ABTS solution and the absorbance was taken at 734 nm after 7 min using a spectrophotometer Aquamate Helion (Thermo Fisher Scientific, Waltham, MA, USA). Distilled water was used as a blank. Measurements were carried out in triplicate for each extract sample. The ABTS· + scavenging was calculated from the Eq. (2):

2 $$ \% {\text{ of ABTS}} \cdot + {\text{ scavenging }} = \, 1 - \frac{As}{{Ac}} \times 100, $$

where: As—absorbance of the sample; Ac—absorbance of the control sample.

#### Determination of intracellular levels of reactive oxygen species (ROS)

In order to determine the ability of the analyzed ferments and YM extract to reduce the intracellular production of reactive oxygen species in keratinocyte and fibroblast cells, a fluorogenic H2DCFDA dye was used. After passive diffusion of this compound into the cells, it is deacetylated by intracellular esterases to a non-fluorescent compound. In the presence of reactive oxygen species it is oxidized and transformed into highly fluorescent DCF^65^. To determine the intracellular level of ROS in HaCaTs and fibroblasts, cells were seeded in 96 well plates at a density of 1 × 10^4^ cells per well. Then, cells were cultured in an incubator for 24 h. DMEM medium was removed and replaced with 10 µM H2DCFDA (Sigma Aldrich, Saint Louis, MO, USA) dissolved in serum free DMEM medium. HaCaT and BJ cells were incubated in H2DCFDA for 45 min and then incubated with YM Kombucha ferments and extract in the concentration of 250 µg/mL. Cells treated with 1 mM hydrogen peroxide (H2O2) were used as positive controls. The control samples were cells untreated with the tested extracts. DCF fluorescence was measured every 30 min for 90 min using a FilterMax F5 microplate reader (Thermo Fisher Scientific) at a maximum excitation of 485 nm and emission spectra of 530 nm.

### Cytotoxicity analysis

#### Cell culture

Two skin cell lines were used in the cytotoxicity analyzes of the tested samples. HaCaT cells (normal human keratinocytes) were obtained from the CLS Cell Lines Service (Eppelheim, Germany), while BJ cells (fibroblasts, ATCC^®^CRL-2522™) were purchased from the American Type Culture Collection (Manassas, VA, USA). Cells were grown in an incubator at 37 °C in a humidified atmosphere of 95% air and 5% carbon dioxide (CO_2_). The research used DMEM (Dulbecco’s Modified Eagle Medium, Biological Industries, Cromwell, CO, USA), supplemented with l-glutamine, 4.5 g/l-glucose and sodium pyruvate. To achieve optimal cell growth, the culture medium was additionally supplemented with 10% (v/v) fetal bovine serum (FBS, Biological Industries, Beit-Haemek, Israel) and 1% (v/v) with antibiotics (100 U/mL penicillin and 1000 μg/mL streptomycin, Thermo Fisher Scientific, Waltham, Massachusetts, USA).After the cultured cells (HaCaT and BJ) had reached approximately 70% confluency, they were exposed to the test samples. For this, the DMEM culture medium was removed from the culture flask (VWR, Radnor, PE, USA) and the cells were washed three times with sterile PBS (phosphate buffered saline, Sigma-Aldrich, Saint Louis, Missouri, USA). The cell layer was trypsinized with trypsin/EDTA (Gibco) and then the cells were suspended in fresh DMEM medium. Cells were then plated in 96-well plates and incubated. After attaching HaCaT and fibroblasts to the bottom of the plates, the cells were exposed to 24-h exposure to various concentrations (10, 100, 250, 500 and 1000 µg/mL) of the extract and various types of Yerba Mate ferments (F7, F14, F21, F35). The control was cells grown in DMEM medium without the addition of extract or ferments^66^.

#### Neutral red uptake assay

Neutral Red uptake test the first test used to evaluate the cytotoxicity of the extract and Yerba Mate ferments on HaCaT and BJ cells was the neutral red uptake test (Sigma Aldrich, Poznań, Poland). This test was performed according to the previously described procedure by Borenfreund et al.^67^. Briefly, after 24 h of exposure to the YM extract and the individual ferments (F7, F14, F21 and F35), the culture medium containing various concentrations of the tested samples (10–1000 µg/mL) was aspirated. Then the cells were incubated for 2 h with a neutral red dye (at a concentration of 40 µg/mL) which was dissolved in serum-free DMEM medium. After incubation with the NR dye, cells were washed twice with sterile PBS. Then 150 µL of decolorizing buffer (C_2_H_5_OH/CH_3_COOH/H_2_O, 50%/1%/49%) was added to each well to release the dye from the cells. Cells were shaken for 15 min on a rocker shaker. After shaking, the absorbance of the dissolved dye was measured at λ = 540 nm using a FilterMax F5 Multi-Mode microplate reader (Thermo Fisher). The absorbance of the control cells (untreated with the extract and ferments) was taken as 100% cell viability. As part of the study, three independent experiments were carried out in which each concentration of extracts was tested in three replications. The % of cell viability was calculated from the Eq. (3):

3 $$\text{\% of cell viability }= \frac{As}{Ac} \times 100,$$

where: As—absorbance of the sample; Ac—absorbance of the control sample.

#### Alamar Blue assay

The cytotoxicity assessment was also performed using the Alamar Blue test. For this purpose, the methodology described by Page et al.^68^ was used. After 24 h of exposure of HaCaT and BJ cells to the YM extract and individual ferments (in the concentration range from 10 to 1000 µg/mL), the DMEM culture medium was aspirated. Sterile resazurin (Sigma Aldrich) at a final concentration of 500 mg/mL dissolved in DMEM medium was added to the wells of a 96-well plate with test cells and incubated for 2 h at 37 °C in the dark. In the last step, the fluorescence of the samples was measured at λ = 570 nm using a microplate reader (FilterMax F5, Thermo Fisher). In order to evaluate the cytotoxicity of the extract and the ferments, three independent experiments were performed in which the fluorescence in 4 wells was measured for each concentration of the extract and the ferments. The results were expressed as the percentage of cell viability compared to the control sample (100%) which was cells maintained in DMEM medium. The % of cell viability was calculated from the Eq. (3).

### Assessment of matrix metallopeptidades inhibition

#### Determination of anti-collagenase activity

The ability of the tested samples (F7, F14, F21, F35 and YM extract) to inhibit collagenase activity was analyzed using a fluorometric Collagenase Inhibitor Screening Kit (ab211108, Abcam). The Kombucha ferments and YM extract at the concentration of 500 µg/mL were used for the analysis. According to the manufacturer’s instructions and with the procedure described previously by Nizioł-Łukaszewska et al.^69^, analyses were performed in a standard 96-well plate with a clear flat bottom. Initially, collagenase (COL) was dissolved in a collagenase assay buffer (CAB). The test samples were prepared by mixing obtained ferments and YM extract with COL and CAB. Inhibitor controls were prepared by mixing inhibitor (1,10-phenanthroline (80 mM) with diluted collagenase and CAB buffer. Enzyme controls were prepared by mixing diluted COL with CAB. The CAB buffer was used as background control. The samples were then incubated for 15 min at room temperature. Meanwhile, a reaction mixture was prepared by mixing the collagenase substrate with CAB. The reaction mixture was then added to all prepared samples and mixed thoroughly. Subsequently, the fluorescence was immediately measured at excitation wavelength λ = 490 nm and emission λ = 520 nm using a microplate reader (FilterMaxF5,ThermoFisher). The measurement was performed in the kinetic mode for 60 min at 37 °C. All samples were prepared in duplicate according to the manufacturer’s instructions. The ability to inhibit COL activity by the analyzed samples was calculated from Eq. (4):

4 $$\text{\% relative COL inhibition }= \frac{Enzyme\,control-Sample}{Enzyme\,control}\hspace{0.17em}\times 100.$$

#### Determination of anti-elastase activity

To determine the possibility of inhibiting another matrix metalloproteinase, neutrophil elastase (NE), a fluorometric kit (Abcam, ab118971) was applied. The Kombucha ferments and YM extract at the concentration of 500 µg/mL were used for the analysis. Analysis was performed in 96-well black plates (transparent bottoms). The test procedure was based on the instructions provided by the manufacturer and described by Nizioł-Łukaszewska et al.^69^. Initially, NE enzyme solutions, NE substrate and inhibitor control (SPCK) were prepared according to the instructions. Then, diluted NE solution was added to all wells. Test samples, inhibitor control and enzyme control (Assay Buffer) were added to subsequent wells. All samples were prepared in duplicate. After all reagents were added, the samples were mixed. The plate was then incubated at 37 °C for 5 min. In the meantime, a reaction mixture was prepared by mixing the Assay Buffer and NE substrate. The mixture was added to each well and mixed thoroughly. Fluorescence was measured immediately at excitation wavelength λ = 400 nm and emission λ = 505 nm using a microplate reader (FilterMax F5, Thermo Fisher Scientific, Waltham, MA, USA).The kinetic mode was used (30 min at 37 °C). The ability to inhibit NE activity by the analyzed samples (F7, F14, F21, F35 and YM) was calculated from the Eq. (5):

5 $$ {\text{\% relative }}\;{\text{NE}}\;{\text{ activity }} = { }\frac{\Delta RFU\; test\; inhibitor}{{\Delta RFU\; enzyme \;control}} \times 100. $$

### Assessment of anti-inflammatory potential

#### Inhibition of lipoxygenase activity

The ability of obtained extracts to inhibit lipoxygenase activity was determined using a method described by Sarvesvaran et al.^70^. Then, 10 µL of obtained ferments and YM extract (100, 500 and 1000 µg/mL) were mixed in 96-well plate with 160 µL of 100 mM PBS and 20 µL of soybean lipoxygenase solution (167 U/mL). Solutions were incubated at 25 °C for 10 min and then 10 µL of sodium linoleic acid was added to initiate the reaction. The absorbance was measured at 234 nm over a period of 3 min in every minute using a FilterMax F5microplate reader (Thermo Fisher Scientific, Waltham, MA, USA). Quercetin (100, 500 and 1000 µg/mL) was used as positive control. The final result was the arithmetic mean of three independent measurements. The percent of lipoxygenase activity inhibition was calculated from Eq. (6):

6 $$\text{\% inhibition of lipoxygenase activity }= \frac{Ac-As}{Ac}\times 100,$$

where: As—absorbance of the sample; Ac—absorbance of the control sample.

### Transepidermal water loss (TEWL) and skin hydratation measurements

TEWL and skin hydration measurements were conducted using TEWAmeter TM 300 probe and Corneometer CM 825 probe connected to a MPA adapter (Courage + Khazaka Electronic, Köln, Germany). The study was conducted on 15 volunteers, according to the procedure described by Nizioł-Łukaszewska et al.^71^. Five areas (2 × 2 cm in size) were marked on the forearm skin. 0.2 mL of 100 µg/mL solution of F7, F14, F21, F35 and YM (aqueous solutions of dry ferments and extract) was applied to 5 fields. One field (control field) was not treated with any sample. Sample solutions were gently spread over every skin fragment, and then rinsed with distilled water and dried with a paper towel. After 60, 180 and 360 min, the hydration and TEWL measurements were taken. The final result was the arithmetic mean (from each volunteer) of 5 independent measurements (skin hydration) and 20 measurements (TEWL).

### Statistical analysis

Values of different parameters were expressed as the mean ± standard deviation (SD). The two‐way analysis of variance (ANOVA) and Bonferroni posttest between groups were performed at the level p value of < 0.05 to evaluate the significance of differences between values. Statistical analyses were performed using GraphPad Prism 8.4.3 (GraphPad Software, Inc., San Diego, CA, USA) and Statistica 9.0 (StatSoft, CA, USA) using one‐way ANOVA and Tukey’s test.

### Ethical approval

The study complies with local and national regulations.

## Conclusions

So far, literature data indicate that fermentation by Kombucha is carried out for black and green tea. The results obtained in this study showed that Yerba Mate extract can also be a good substrate for Kombucha fermentation. The presence of active substances, mainly polyphenols, such as chlorogenic acid or caffeoyl derivatives, as well as xanthines and flavonoids, may indicate a high antioxidant potential in both YM extract and Kombucha ferments. Fermented YM extracts showed a positive effect on skin cells: keratinocytes and fibroblasts. Both YM extract and Kombucha ferments have anti-aging and anti-inflammatory properties, which is extremely important in the context of healthy skin appearance and prevention of various skin imperfections. Comparing the fermentation time of Kombucha with the use of Yerba Mate extract as a fermentation medium, the ferment showed the most favorable properties after 14 and 21 days of fermentation. The obtained results indicate that the ferments, apart from their probiotic activity supporting the beneficial microorganisms inhabiting the human skin, can also be a valuable ingredient present in pharmaceutical and cosmetic products.
